# Association between blood glucose levels and arterial stiffness marker: comparing the second derivative of photoplethysmogram and cardio-ankle vascular index scores

**DOI:** 10.3389/fendo.2023.1237282

**Published:** 2023-09-21

**Authors:** Young-Jae Park

**Affiliations:** ^1^ Department of Biofunctional Medicine & Diagnostics, College of Korean Medicine, Kyung Hee University, Seoul, Republic of Korea; ^2^ Department of Diagnosis and Biofunctional Medicine, Kyung Hee University Hospital at Gangdong, Seoul, Republic of Korea

**Keywords:** CAVI, SDPTG, arterial stiffness, hierarchical regression model, Korean population

## Abstract

**Objective:**

This study aimed to compare the association between fasting plasma glucose (FPG) and glycosylated hemoglobin A1c (HbA1c) levels using the second derivative of photoplethysmogram (SDPTG) index and the cardio-ankle vascular index (CAVI).

**Methods:**

Electronic medical records of 276 participants (160 men, 116 women) who visited the health promotion center of a university hospital were examined. Age, sex, body mass index (BMI), blood pressure, and lipid profile were considered as risk factors for arterial stiffness, together with the FPG, HbA1c, CAVI, and SDPTG indices. Hierarchical regression models were constructed, and all participants were divided into low-normal, high-normal, prediabetic, and diabetic groups to examine the group-based differences in CAVI and SDPTG indices.

**Results:**

FPG and HbA1c were independently predictive of increased CAVI, and their predictive powers for CAVI were equivalent (*β* = 0.214 and 0.200, respectively). Risk factors, including age, BMI, and male sex, were also predictive of CAVI (*β*= 0.593-0.630, -0.256 – -0.280, and 0.142-0.178, respectively). None of the FPG and HbA1c values were predictive of the SDPTG indices. The CAVI was higher in the diabetes group than in the other three groups according to HbA1c level, while the d/a index of the SDPTG decreased in the prediabetes group and increased in the diabetes group.

**Conclusions:**

CAVI may not be substituted for SDPTG indices when evaluating arterial stiffness based on the glucose level. Moreover, the progression rate of arterial stiffness may differ between the diabetic and nondiabetic stages.

## 1 Introduction

Increased fasting plasma glucose (FPG) levels are risk factors for arterial stiffness and cardiovascular disease (CVD) (1). Compared with FPG, glycosylated hemoglobin A1c (HbA1c) is a more stable indicator of glucose levels over the previous 3−4 months (2). Similar to FPG, an increase in HbA1c has been reported to be related to the development of arterial stiffness (3). Arterial stiffness can be noninvasively assessed using the pulse wave velocity (PWV). Carotid-femoral PWV (cf-PWV) is recommended as a representative marker for cardiovascular risk in individuals with hypertension by the European Society of Cardiology and the American Heart Association (4, 5). Together with cf-PWV, brachial-ankle PWV (ba-PWV) and cardio-ankle vascular index (CAVI) have been widely used to examine the severity of arterial stiffness because of the convenience of measurement (6–8). Some studies have suggested that increased FPG or HbA1c is related to arterial stiffness estimated by ba-PWV and CAVI not only in patients with diabetes (9, 10), but also in participants with normal and high-normal glucose levels (8, 11). These results suggest a graded increase in blood glucose levels with worsening arterial stiffness, irrespective of the diagnosis of diabetes.

The second derivative of the photoplethysmogram (SDPTG) is another indicator of arterial stiffness (12), and is related to risk factors of CVD, including hypertension and dyslipidemia (13). In terms of convenience, SDPTG measurement is much simpler than ba-PWV and CAVI measurement because the former requires only one photoplethysmography (PTG) transducer attached to the participant’s index finger and completes in less than 2 min. Although the relationship between the increase in ba-PWV and CAVI and the increase in blood glucose level is well established, studies on the relationship between blood glucose level and arterial stiffness using SDPTG are controversial. One study reported that the ratio of the amplitude of the b wave to the a wave of the SDPTG (b/a) was higher in patients with diabetes with HbA1c ≥ 8.0% than that in patients with diabetes with HbA1c < 8.0% (14). Another study reported a non-significant relationship between SDPTG indices and blood glucose levels in a large population (15). To examine whether SDPTG indices reflect arterial stiffness based on rising glucose levels, it may be helpful to simultaneously utilize SDPTG and CAVI for a population, including normal, prediabetic, and diabetic groups. Previously, Bortolotto et al. examined SDPTG and cf-PWV in patients with hypertension and found that the blood glucose level was predictive only of cf-PWV, not SDPTG indices (15). However, none of the studies have compared SDPTG with CAVI. This study mainly aimed to comparatively assess the relation of SDPTG indices and CAVI as a marker of arterial stiffness based on the increase in FPG and HbA1c levels. Previous studies have reported significant aging effects and sex differences in FPG and HbA1c levels in CAVI (5, 16). SDPTG has been also reported to have an aging effect (12, 13). However, standardized beta (*β*) coefficients were not reported in previous studies; therefore, it was not possible to compare the strength of the influence of FPG or HbA1c on other risk factors. In this study, a hierarchical regression model was constructed, and in addition to unstandardized beta values, *β* values of FPG, HbA1c, and other risk factors were examined.

Even if a linear relationship between FPG, HbA1c, and arterial stiffness is widely accepted, there is still a possibility that arterial stiffness may change suddenly or fluctuate among the normal, prediabetic, and diabetic groups. Gomez-Sanchez et al. reported that CAVI in subjects with high glucose levels was higher than that in subjects with normal and high-normal glucose levels in a Caucasian population (17). Shin et al. reported significant differences in ba-PWV between groups according to FPG levels in a Korean population (8). However, no study has addressed the differences in the SDPTG and CAVI indices according to glucose levels in a Korean population. Therefore, this study also aimed to examine the differences in CAVI and SDPTG indices according to FPG and HbA1c levels in a Korean population.

## 2 Methods

### 2.1 Subjects

This study followed a cross-sectional design. Among the 466 subjects who visited the health promotion center of a university hospital between April 2014 and December 2014, electronic medical recordings (EMRs) of 276 subjects who completed CAVI, SDPTG, FPG, HbA1c, and lipid profiles were investigated. Data on FPG, HbA1c, total cholesterol, and high- and low-density lipoprotein cholesterol (HDL- and LDL-cholesterol) levels after an overnight fast were collected from the EMRs of all subjects. Data on blood samples, the heart rate (HR), systolic and diastolic blood pressure (SBP and DBP), and body mass index (BMI) were collected. The CAVI and SDPTG tests were conducted in random order. The study protocol was approved by the Institutional Review Board of the Kyung Hee University Korean Medical Hospital at Gangdong (IRB approval number: KHNMCOH 2016-09-002-002).

### 2.2 CAVI and SDPTG measurements

The CAVI was measured using a VS-1000 (Fukuda Denshi, Tokyo, Japan) with a supine position. This device includes four cuffs for estimating the BP and obtaining the PTG data of the left and right brachial and tibial arteries. Further, the phonocardiograms and electrocardiogram transducers were attached to the participants’ left chest. Through the 5 minute-test, the left and right CAVI values were automatically calculated. Although PTG data were recorded from the brachial and tibial arteries, only the PTG data of the brachial artery were used to estimate the time point of the first cardiac sound, which was caused by the opening of the aortic valve, and this time point corresponding to that at which the PTG for the tibial artery was generated. The stiffness parameter *S* was estimated by the following equation: *S* = 2ρ × 1/(Ps − Pd) × ln (Ps/Pd) × PWV^2^, where ρ is blood density, and Ps and Pd are SBP and DBP in mmHg, respectively (16). For the SDPTG measurement, each participant was seated comfortably in a chair, and the PTG was recorded for 90-second periods on the index finger of the left hand using SA-3000P (Medicore Co., Seoul, Korea). The SDPTG consists of four systolic waves (a, b, c, and d) and a diastolic wave (e). The b/a reflects large artery stiffness, whereas the ratio of the amplitude of the d wave to the a wave (d/a) reflects an earlier-returned component of the pulse wave caused by peripheral artery stiffness (12). The vascular aging index (VAI = (b-c-d-e)/a) reflects both large and peripheral artery stiffness (15). Since SA-3000P did not present e-wave information, a modified VAI (mVAI = (b-c-d)/a) was considered in this study (18).

### 2.3 Formation of the hierarchical regression model

Since there was a very strong correlation between the left and right CAVIs (r = 0.954), the mean left-right CAVI value was used during regression analysis. The association of aging (10, 12, 13), sex differences (8, 15), obesity (19), hypertension (15, 20), and dyslipidemia (21) with arterial stiffness has been previously reported. While previous studies used multiple regression models to calculate the predictability of risk factors and glucose level indicators for arterial stiffness (5, 6, 8, 11), this study adopted hierarchical regression models consisting of two blocks of independent variables. In the hierarchical regression model, risk factors including age, sex differences, BMI, BP, and lipid-related markers were inserted into the first block, and the overall predictive power of the risk factors for arterial stiffness was calculated. One of the FPG or HbAlc was inserted into the second block, and the influence of FPG or HbA1c on arterial stiffness was calculated. The reason for separating FPG and HbA1c from the second block was that these two indicators had the possibility of multicollinearity. After designing the two blocks of independent variables, three SDPTG indices (mVAI, b/a, and d/a) and CAVI were used as dependent variables in the regression models. Because one of the FPG or HbA1c levels was input to the dependent variable, a total of eight hierarchical regression models were formed. On comparing the *β*-values of FPG and HBA1c with the *β*-values of the other risk factors, the eight hierarchical regression models yielded information on the comparative predictability of FPG and HBA1c with other risk factors on arterial stiffness, as well as the comparative predictability of FPG with HBA1c on arterial stiffness.

### 2.4 Classification of normal, prediabetes, and diabetes groups

To examine differences in CAVI and SDPTG indices according to FPG and HbA1c levels, all subjects were categorized into four groups: low-normal, high-normal, prediabetes, and diabetes^8^. Using the diagnostic criteria of the American Diabetes Association by FPG (22), 17 subjects with FPG > 126 mg/dL were categorized into the diabetes group. The remaining 259 subjects were categorized into three groups according to tertiles of FPG levels: low-normal (n=84, FPG<89 mg/dL), high-normal (n=86, 89 mg/dL ≤ FPG <98 mg/dL), and prediabetes (n = 89, 98 mg/dL ≤ FPG<126 mg/dL) groups. Similar to the FPB classification, four groups were defined according to HbA1c levels. That is, 20 subjects with HbA1c over 6.5% were categorized into the diabetes group (22), and the remaining 256 subjects were assigned to one of the three groups by HbA1c levels: low-normal (n=89, HBA1c < 5.3%), high-normal (n=95, 5.3%≤ HbA1c <5.6%), and prediabetes (n=72, 5.6% ≤ HbA1c <6.5%) groups. In this study, the four groups based on FPG and HbA1c levels were abbreviated as quartile 1 (Q1, low-normal), Q2 (high-normal), Q3 (prediabetes), and Q4 (diabetes), although the number of patients in the diabetes group was smaller than that in the other three groups.

### 2.5 Statistical analysis

In the hierarchical regression model, multivariate normality for age, HR, SDP, DBP, BMI, FPG, and HbA1c values were checked using the Kolmogorov−Smirnov test. If any indices violated normality, they were log-transformed. All the independent variables were inserted into blocks using a stepwise method. The significance of the F-value was investigated to examine whether the significance of the new regression model remained when one independent variable was added. A variance inflation factor (VIF) was used to examine whether there was any multicollinearity between the independent variables. If there were aging effects on CAVI and SDPTG indices, partial correlations with age as a covariate were conducted for CAVI, mVAI, b/a, and d/a values. Partial correlations were compared with the results of Pearson or Spearman correlations to examine how the aging effect resulted in changes in the correlations between the CAVI and SDPTG indices.

If FPG or HbA1c was predictive of any indices of CAVI or SDPTG, differences in CAVI or SDPTG indices between FPG or HbA1c groups were investigated to examine which factor, among FPG or HbA1c, was more effective for changes in arterial stiffness levels. If the regression modeling found age effects and sex differences, a two-way analysis of covariance (ANCOVA) with age as a covariate was considered. In the ANCOVA model, sex and the four groups according to FPG or HbA1c levels were assigned to two independent factors. However, the sample numbers of the four groups were not equal, so equality of error variances needed to be guaranteed before the ANCOVA test. Therefore, a two-way ANCOVA was conducted only if the equality of error variances was accepted. If equality of error variances was violated, differences in CAVI or the SDPTG indices by FPG or HbA1c groups were examined using the Kruskal−Wallis rank test and differences between two groups and between sexes within each group were examined using the Mann−Whitney U test. All statistical analyses were conducted using Statistical Package for Social Sciences version 21 (SPSS, Inc., Chicago, Illinois, USA). Values are presented as means ± standard deviations, and *P* values < 0.05 were considered statistically significant. In the hierarchical regression models, a VIF above 10 denoted multicollinearity between independent variables (23).

## 3 Results

**Table 1** lists sex-based descriptive characteristics of age, FPG, HbA1c, BP, and BMI. In the multivariate normality test, HR, HDL cholesterol, FPG, and HbA1c values were not normally distributed, and these values were log-transformed when inserted into the hierarchical regression model. **Table 2** lists the regression model summary of SDPTG and CAVI as the dependent variables. In the models with mVAI, b/a, and d/a as dependent variables, none of FPG and HbA1c contributed to an increase in the adjusted R^2^ values of mVAI, b/a, and d/a (significance of F change: 0.679 -0.836), although age, sex differences, BMI, DBP, and LDL-cholesterol independently contributed to an increase in the adjusted R^2^ values of the SDPTG indices (significance of F change: 0.0001 -0.037). This indicated that FPG and HbA1c levels were not predictive of any SDPTG index. In the models with CAVI as the dependent variable, FPG and HbA1c contributed to the increase in adjusted R^2^ values (significance of F change < 0.001), indicating that FPG and HbA1c were independent predictors of arterial stiffness estimated by CAVI, even after controlling for risk factors such as age, sex, and BMI. The adjusted R^2^ values of age, sex differences, and BMI in the CAVI model were approximately twice those in the SDPTG model, indicating that the predictive power of risk factors to CAVI was stronger than that to the SDPTG indices.

**Table 1:** Sex-based descriptive characteristics of study participants.

	Sex	Men (n=160)			Women (n=116)		
		Mean ± SD	Minimum	Maximum	Mean ± SD	Minimum	Maximum
	Age (years)	52.53 ± 7.51	22	71	51.67 ± 7.85	31	77
Glucose	FPG (mg/dL)	102.36 ± 19.11	72.00	198.00	91.34 ± 18.47	63.00	245.00
	HbA1c (%)	5.62 ± 0.68	4.60	9.80	5.44 ± 0.72	4.80	10.50
CAVI	Lt. CAVI	7.73 ± 0.85	5.90	10.20	7.44 ± 0.78	5.40	9.50
	Rt. CAVI	7.81 ± 0.87	5.90	10.20	7.50 ± 0.78	5.40	9.20
	Mean CAVI	7.77 ± 0.85	5.90	10.10	7.47 ± 0.77	5.40	9.25
SDPTG	mVAI (ratio)	-59.98 ± 34.78	-143.79	46.30	-46.18 ± 40.93	-151.55	75.71
	b/a (ratio)	-81.63 ± 16.47	-124.52	-19.18	-73.51 ± 18.02	-118.81	-11.47
	d/a (ratio)	-35.91 ± 15.64	-82.87	-4.59	-38.54 ± 16.33	-129.20	-13.34
Hemodynamic	HR (beats per minute)	64.08 ± 9.11	42.00	112.00	62.37 ± 8.26	43.00	88.00
	SBP (mmHg)	120.93 ± 10.96	91.00	160.00	114.90 ± 12.08	89.00	161.00
	DBP (mmHg)	75.77 ± 7.55	56.00	96.00	70.03 ± 8.32	47.00	89.00
Obesity	BMI (kg/m^2^)	24.97 ± 2.67	18.60	35.10	23.28 ± 3.48	18.00	40.50
Lipid profile	Total cholesterol (mg/dL)	195.18 ± 37.63	83.00	298.00	199.87 ± 37.45	119.00	305.00
	HDL-cholesterol (mg/dL)	51.88 ± 11.65	30.00	92.00	60.23 ± 15.22	29.00	101.00
	LDL-cholesterol (mg/dL)	136.99 ± 33.08	52.00	243.00	136.00 ± 32.52	70.00	225.00

FPG, fasting plasma glucose; HbA1c, glycosylated A1c; CAVI, cardio-ankle vascular index; SD, standard deviation; Lt, left; Rt, right; SDPTG, second derivative of photoplethysmogram; mVAI, modified vascular index; HR, heart rate; SBP, systolic blood pressure; DBP, diastolic blood pressure; BMI, body mass index; HDL, high-density lipoprotein; LDL, low-density lipoprotein.

**Table 2:** Regression model summary with CAVI and SDPTG indices as dependent variables.

Dependent variable		Model	Adjusted R^2^	Independent variable	P-value	F-change	Significance of F change
SDPTG	mVAI	Model 1	0.190	Age	<0.001	65.709	<0.001
		Model 1	0.230	Age, Sex	<0.001	41.967	<0.001
		Model 1	0.240	Age, Sex, BMI	<0.001	30.008	0.028
		Model 2 (FPG)	0.238	Age, Sex, BMI, FPG	<0.001	0.043	0.836
		Model 2 (HbA1c)	0.238	Age, Sex, BMI, HbA1c	<0.001	0.019	0.890
	b/a	Model 1	0.135	Age	<0.001	43.916	<0.001
		Model 1	0.195	Age, Sex	<0.001	34.21	<0.001
		Model 1	0.212	Age, Sex, HR	<0.001	25.707	0.008
		Model 1	0.222	Age, Sex, HR, BMI	<0.001	20.62	0.037
		Model 2 (FPG)	0.220	Age, Sex, HR, BMI, FPG	<0.001	0.172	0.679
		Model 2 (HbAlc)	0.219	Age, Sex, HR, BMI, HbA1c	<0.001	0.067	0.796
	d/a	Model 1	0.134	Age	<0.001	43.401	<0.001
		Model 1	0.187	Age, HR	<0.001	32.606	<0.001
		Model 1	0.209	Age, HR, DBP	<0.001	23.433	<0.001
		Model 1	0.220	Age, HR, DBP, LDL-cholesterol	<0.001	18.881	0.038
		Model 2 (FPG)	0.227	Age, HR, DBP, LDL-cholesterol, FPG	<0.001	3.583	0.059
		Model 2 (HbAlc)	0.219	Age, HR, DBP, LDL-cholesterol, HbA1c	<0.001	0.670	0.414
CAVI		Model 1	0.387	Age	<0.001	174.464	<0.001
		Model 1	0.415	Age, BMI	<0.001	14.200	<0.001
		Model 1	0.453	Age, BMI, Sex	<0.001	19.956	<0.001
		Model 1	0.460	Age, BMI, Sex, HR	<0.001	4.4.38	0.036
		Model 2 (FPG)	0.497	Age, BMI, Sex, HR, FPG	<0.001	21.305	<0.001
		Model 2 (HbAlc)	0.494	Age, BMI, Sex, HR, HbA1c	<0.001	19.271	<0.001

CAVI, cardio-ankle vascular index; SDPTG, second derivative of the photoplethysmogram; mVAI, modified vascular aging index (mVAI = (b-c-d)/a), FPG, fasting plasma glucose; HR, heart rate; BMI, body mass index; DBP, diastolic blood pressure; LDL-cholesterol, low-density lipoprotein cholesterol. In each hierarchical model, only one among the FPG and HbA1c was separately inserted in the second block of independent variables.

**Table 3** lists unstandardized and *β* values, standard errors, and VIF values of the independent variables in the regression models. All independent variables showed the VIF levels under two points, indicating that they were free of multicollinearity. In the comparison of *β* values, age was the most influential factor, ranging from -0.327 to 0.630. In the two regression models with CAVI as a dependent variable, *β* value of FPG was 0.214, being similar to 0.200 of HbA1c. The *β* values of the sex variable in the two CAVI models were -0.142 (FPG model) and -0.178 (HbA1c model), indicating that CAVI was higher in men than women. Inversely, *β* value of sex in the b/a model was 0.206, indicating that b/a was higher in women than men. The *β* values of BMI in the two CAVI models were -0.280 (FPG model) and -0.256 (HbA1c model), indicating that lower BMI was associated with increased arterial stiffness. Although final regression models of the CAVI were significant (P <0.001), the P value of HR exceeded 0.05 when FPG or HbA1c was inserted as an independent variable. This indicated the possibility of a decrease in HR according to an increase in FPG or HbA1c.

**Table 3:** Model summary of hierarchical regression with CAVI and SDPTG indices as dependent variables.

Index	Dependent variable (final adjusted R^2^)	Independent variable	Unstandardized beta value (B)	Standard error	Standardized beta value (*β*)	*t* value	*P* value	95% CI	Tolerance	VIF
SDPTG	mVAI (0.240)	Age	2.301	0.263	0.463	8.748	<0.001	1.783-2.819	0.987	1.013
		Sex	13.309	4.195	0.173	3.173	0.002	5.051-21.567	0.929	1.077
		BMI	-1.467	0.663	-0.121	-2.212	0.028	-2.774–0.161	0.920	1.087
	b/a (0.222)	Age	0.878	0.124	0.382	7.079	<0.001	0.634-1.122	0.970	1.031
		Sex	7.332	1.965	0.206	3.732	<0.001	3.465-11.200	0.926	1.080
		HR	-16.544	7.092	-0.127	-2.333	0.020	-30.505–2.582	0.954	1.048
		BMI	-0.658	0.314	-0.118	-2.097	0.037	-1.277–0.040	0.898	1.114
	d/a (0.206)	Age	-0.682	0.114	-0.327	-5.978	<0.001	-0.906–0.457	0.966	1.035
		HR	29.577	6.501	0.250	4.549	<0.001	16.777-42.377	0.955	1.047
		DBP	-0.252	0.106	-0.132	-2.373	0.018	-0.460–0.043	0.933	1.071
		LDL-cholesterol	0.055	0.027	0.114	2.088	0.038	0.003-0.108	0.969	1.032
CAVI	CAVI^a^ (0.497)	Age	0.068	0.005	0.630	14.441	<0.001	0.059-0.078	0.961	1.041
		Sex	-0.238	0.077	-0.142	-3.079	0.002	-0.391–0.086	0.855	1.169
		BMI	-0.074	0.012	-0.280	-6.125	<0.001	-0.098–0.050	0.872	1.147
		HR	0.491	0.270	0.080	1.821	0.070	-0.040-1.021	0.948	1.054
		FPG	1.056	0.229	0.214	4.616	<0.001	0.606-1.507	0.846	1.182
	CAVI^b^ (0.494)	Age	0.64	0.005	0.593	13.038	<0.001	0.055-0.074	0.891	1.123
		Sex	-0.298	0.075	-0.178	-3.968	<0.001	-0.447–0.150	0.913	1.095
		BMI	-0.067	0.012	-0.256	-5.636	<0.001	-0.091–0.044	0.895	1.117
		HR	0.474	0.271	0.077	1.751	0.081	-0.059-1.008	0.946	1.058
		HbA1c	1.536	0.350	0.200	4.390	<0.001	0.847-2.226	0.885	1.129

CAVI, cardio-ankle vascular index; SDPTG, second derivative of the photoplethysmogram; VIF, variance inflation factor; mVAI, modified vascular aging index; FPG, fasting plasma glucose; Sex, men, 1: women, 2; HR, heart rate; BMI, body mass index; DBP, diastolic blood pressure; LDL-cholesterol, low-density lipoprotein cholesterol; CI, confidence interval.

a regression model with FPG as an independent variable,

b regression model with HbA1c as an independent variable. In the regression analysis, HR, FPG, and HbA1c were log-transformed.

As the right CAVI did not satisfy multivariate normality using the Kolmogorov–Smirnov test (z = 1.552, P=0.016), Spearman’s correlations between CAVI and the SDPTG indices were calculated. Moreover, regression analysis results showed a strong age effect on the SDPTG indices and CAVI, so a partial correlation with age as a covariate was conducted. **Table 4** lists the Spearman and partial correlations between the CAVI and SDPTG indices. Spearman’s correlations showed moderate positive or negative correlations between the CAVI and SDPTG indices (r = ± 0.319 – ± 0.410). However, partial correlations between them were notably decreased compared to Spearman’s correlations, without controlling for age. Moreover, the correlation between CAVI and the b/a index became non-significant in the partial correlations.

**Table 4:** Spearman- and partial- correlations between CAVI and the SDPTG indices.

Correlation	SDPTG	CAVI		
		Lt. CAVI	Rt. CAVI	Mean CAVI
Spearman’s correlation	mVAI	0.388^**^	0.373^**^	0.387^**^
	b/a	0.332^**^	0.319^**^	0.334^**^
	d/a	-0.390^**^	-0.410^**^	-0.406^**^
Partial correlation	mVAI	0.145^*^	0.127^*^	0.139^*^
	b/a	0.082	0.079	0.082
	d/a	-0.180^**^	-0.211^**^	-0.199^**^

CAVI; cardio-ankle vascular index, SDPTG; second derivative of the photoplethysmogram,

Lt, left; Rt, right; mVAI, modified vascular aging index ((b-c-d)/a). **: P< 0.01, *: P< 0.05.

Differences in CAVI values among the four groups according to FPG and HbA1c levels were examined. As age effect and sex differences for the CAVI were found, a two-way ANCOVA was performed, where four groups and sex were assigned to independent variables, and age was inserted as a covariate. To conduct ANCOVA, it is necessary to guarantee equality of error variance for the dependent variables by the group. As the null hypothesis of equality of variances was accepted for HbA1c (P = 0.185), group effects and sex differences were examined by ANCOVA, and multiple comparisons were performed to examine the differences in HbA1c levels between the groups and between sexes. For FPG groups, the null hypothesis of equality of variances was not accepted (F=2.109, P = 0.043), the group effect of FPG on CAVI was examined using the Kruskal−Wallis rank test and differences between the groups and between the sexes within each group were examined using the Mann−Whitney U test. **Table 5** lists the descriptive characteristics of the CAVI according to the four groups based on HbA1c and FPG. Since two-way ANCOVA was conducted only for the four groups by HbA1c level, age, sex, and group effects, and interaction of sex and group effect on CAVI values are also presented only to the groups by HbA1c. For the groups by FPG level, only the group effect, except for age, sex, and the interaction of sex and age, is presented. The four groups by HbA1c showed a significant age effect (F=133.876, P < 0.001), group effect (F = 4.483, P = 0.004), and sex differences (F=4.391, P=0.037). There was no interaction between sex and group effects (F=0.105, P=0.957). Similar to the HbA1c group, the FPG group showed a significant group effect (χ^2 = ^21.549, P <0.001).

**Table 5:** Differences in the CAVI values between low-normal, high-normal, prediabetes, and diabetes groups by HbA1c and FPG level.

Factor	Group (n=276)	CAVI		Effect	*F or Z* value	*P* value
		Men (n=160)	Women (n=116)			
HbA1c	Low-normal (n=89)	7.40 ± 0.63	7.17 ± 0.70	Age	133.876 (*F*)	**<0.001**
	High-normal (n=95)	7.72 ± 0.78	7.51 ± 0.67	Sex	4.391(*F*)	**0.037**
	Prediabetes (n=72)	7.89 ± 0.93	7.87 ± 0.83	Group	4.483 (*F*)	**0.004**
	Diabetes (n=20)	8.59 ± 0.74	8.18 ± 0.37	Sex*Group	0.105 (*F*)	0.957
FPG	Low-normal (n=84)	7.59 ± 0.78	7.29 ± 0.73	Group	-3.204 (*z*)	**<0.001**
	High-normal (n=86)	7.77 ± 0.85	7.53 ± 0.71			
	Prediabetes (n=89)	7.71 ± 0.83	7.74 ± 0.89			
	Diabetes (n=17)	8.45 ± 0.83	8.43 ± 0.32			

CAVI, cardio-ankle vascular index; FPG, fasting plasma glucose; HbA1c, glycosylated hemoglobin A1c. Significant P values are represented in bold letters.

**Figure 1:**
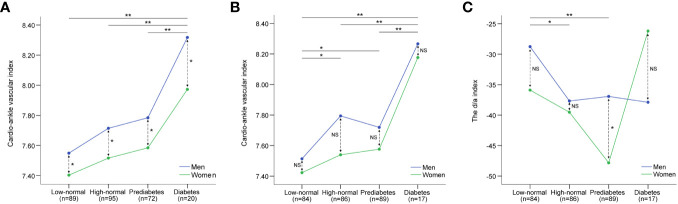
Differences in CAVI and d/a index of SDPTG between low-normal, high-normal, prediabetes, and diabetes groups by HbA1c and FPG levels. CAVI, cardio-ankle vascular index; SDPTG, the second derivative of photoplethysmogram; HbA1c, glycosylated haemoglobin A1c; FPG, fasting plasma glucose. **(A)** differences in CAVI by HbA1c, **(B)** differences in CAVI by FPG, **(C)** differences in d/a index by FPG. Solid arrows indicate comparison between groups, while dotted arrows indicate comparison between sexes. **; P<0.01, *; P<0.05, NS, non-significant.

**Figure 1A** shows the differences in CAVI among the four groups based on HbA1c levels. Multiple comparisons showed that a significant group effect was caused by the differences between one group consisting of the low-normal (Q1), high-normal (Q2), and prediabetes groups (Q3), and the other group consisting of the diabetes group (Q4). That is, CAVI gradually increased in the low-normal, high-normal, and prediabetes groups but suddenly increased in the diabetes group (Q4). Moreover, the CAVI was higher in men than in women in all four groups. **Figure 1B** shows the differences in CAVI among the four groups according to FPG. The Mann-Whitney U test was conducted for six pairs of groups by FPG level (Q1-Q2, Q1-Q3, Q1-Q4, Q2-Q3, Q2-Q4, and Q3-Q4), and for four pairs of sexes within each group (men: women within Q1, Q2, Q3, and Q4). The CAVI of the four groups by FPG showed a step-like difference. That is, the CAVI of the low-normal group was lower than that of the high-normal and prediabetes groups, whereas the CAVI of the high-normal and prediabetes groups was lower than that of the diabetes group. There were no sex differences in the CAVI within each group according to FPG.

Although there was no significant linear relationship between FPG, HbA1c, and the SDPTG indices, it is still possible that there will be differences in mVAI, b/a, and d/a indices between the groups based on FPG and HaA1c. **Table 6** lists the differences in the mVAI, b/a, and d/a indices between the FPG and HbA1c groups. The null hypothesis of equality of error variance was not accepted in the examination of differences in the d/a index by FPG groups, and the group differences were examined using the Kruskal-Wallis rank test. The other SDPTG indices satisfied the equality of error variances, and the group effect and sex differences for the indices were examined using two-way ANCOVA with age as a covariate. None of the SDPTG indices, except for the d/a index by the FPG group, showed significant group effects. Therefore, for the d/a index by FPG groups, the Mann-Whitney U test was conducted for six pairs of groups by and for four pairs of sexes within each group, similar to the examination of differences in CAVI by FPG groups. **Figure 1C** shows the differences in the d/a index between the four groups according to FPG. Similar to the CAVI by FPG groups, the d/a index showed step-like differences between the four groups by FPG. However, d/a in the low-normal group decreased in the high-normal and prediabetes groups and increased again in the diabetes group. Finally, there were no differences in the d/a values between the low-and diabetic groups. Regarding sex differences, the d/a index was higher in men than in women only in the prediabetes group; there were no sex differences among the other three groups.

**Table 6:** Differences in SDPTG indices between low-normal, high-normal, prediabetes, and diabetes groups by HbA1c and FPG level.

SDPTG	Factor	Group (n=276)	Men (n=160)	Women (n=116)	Effect	*F or χ* ^2^ value	*P* value
mVAI	HbA1c	Low-normal (n=89)	-69.78 ± 27.01	-58.37 ± 45.76	Age	53.771 (*F*)	**<0.001**
		High-normal (n=95)	-60.02 ± 31.85	-43.44 ± 34.82	Sex	5.448(*F*)	**0.020**
		Prediabetes (n=72)	-56.92 ± 39.78	-26.69 ± 36.15	Group	0.898 (*F*)	0.443
		Diabetes (n=20)	-43.34 ± 41.14	-47.84 ± 21.03	Sex*Group	0.627 (*F*)	0.598
	FPG	Low-normal (n=84)	-60.42 ± 38.04	-55.36 ± 38.69	Age	67.079 (*F*)	**<0.001**
		High-normal (n=86)	-60.21 ± 34.06	-41.33 ± 43.45	Sex	2.382(*F*)	0.124
		Prediabetes (n=89)	-60.81 ± 32.71	-28.62 ± 37.26	Group	1.476 (*F*)	0.221
		Diabetes (n=17)	-54.50 ± 42.14	-64.99 ± 12.19	Sex*Group	0.981 (*F*)	0.402
b/a	HbA1c	Low-normal (n=89)	-85.75 ± 13.47	-79.42 ± 19.99	Age	40.058 (*F*)	**<0.001**
		High-normal (n=95)	-80.18 ± 16.39	-70.19 ± 16.09	Sex	12.324(*F*)	**0.001**
		Prediabetes (n=72)	-79.89 ± 18.86	-67.99 ± 15.12	Group	1.453 (*F*)	0.228
		Diabetes (n=20)	-80.95 ± 15.78	-71.20 ± 13.38	Sex*Group	0.115 (*F*)	0.951
	FPG	Low-normal (n=84)	-79.95 ± 19.72	-76.86 ± 16.98	Age	47.976 (*F*)	**<0.001**
		High-normal (n=86)	-81.50 ± 17.72	-71.69 ± 19.45	Sex	4.531(*F*)	**0.034**
		Prediabetes (n=89)	-81.98 ± 14.32	-67.04 ± 16.84	Group	1.063 (*F*)	0.365
		Diabetes (n=17)	-83.84 ± 16.31	-82.28 ± 4.24	Sex*Group	0.659 (*F*)	0.578
d/a	HbA1c	Low-normal (n=89)	-30.75 ± 13.72	-34.56 ± 14.94	Age	32.655 (*F*)	**<0.001**
		High-normal (n=95)	-35.56 ± 16.06	-40.42 ± 18.96	Sex	0.054(*F*)	0.816
		Prediabetes (n=72)	-38.23 ± 15.26	-44.33 ± 13.32	Group	1.154 (*F*)	0.328
		Diabetes (n=20)	-43.65 ± 16.80	-31.40 ± 3.61	Sex*Group	1.223 (*F*)	0.302
	FPG^a^	Low-normal (n=84)	-29.64 ± 12.47	-34.34 ± 12.07			
		High-normal (n=86)	-37.44 ± 18.55	-39.46 ± 14.82	Group	8.342 (*χ* ^2^)	**0.039**
		Prediabetes (n=89)	-36.84 ± 13.96	-49.76 ± 24.78			
		Diabetes (n=17)	-40.03 ± 17.97	-29.08 ± 3.31			

a Assumption of equality of error variance was not accepted (P=0.039) and Kruskal-Wallis rank test was applied. SDPTG; second derivative of photoplethysmogram, mVAI; modified vascular aging index, FPG; fasting plasma glucose, HbA1c; glycosylated hemoglobin A1c. Significant P values are represented as bold numbers.

## 4 Discussion

The main finding of this study was that none of the FPG and HbA1c levels were predictive of the SDPTG indices. Nonetheless, the mVAI, b/a, and d/a indices of the SDPTG were predicted by risk factors for arterial stiffness, including age, sex, BMI, DBP, and LDL-cholesterol. In contrast, FPG and HbA1c were predictive of an increase in CAVI, consistent with previous study results (6–11). Another finding was that the CAVI level was higher in the diabetes group than in the low-normal, high-normal, and prediabetes groups by HbA1c level. These findings suggest that CAVI may not be substituted for SDPTG indices when evaluating arterial stiffness according to the increase in glucose level, despite the measurement convenience of SDPTG, and there is a possibility of a difference in the progress speed of arterial stiffness in the diabetic and non-diabetic stages.

Previous studies have reported the relationship between glucose levels and arterial stiffness in normal, prediabetic, and diabetic stages using ba-PWV (5, 8, 11) or CAVI (17). The results of this study support the hypothesis that normal- and prediabetes-glucose levels may result in vascular dysfunction before the diagnosis of diabetes (8), and glucose levels estimated by FPG or HbA1c should be cautiously monitored to prevent the progression of arterial stiffness in the normal and prediabetes stages, as well as in diabetes. This study also supports the study by Gomez-Sanchez, where FPG and HbA1c levels were independently predictive of CAVI (17). However, this study overcomes the limitations of the previous study by using an unstandardized beta coefficient and shows that the association of FPG with CAVI is equivalent to that of HbA1c with CAVI. Although HbA1c has the advantage of being free of fasting and a more stable indicator than FPG, it also has some issues in that the former is affected by the variability of the test method, abnormal hemoglobin, anemia, and racial differences (24). Therefore, it is recommended that the blood glucose test be selected based on the laboratory conditions and the subject’s circumstances.

In the examination of the predictive power of FPG, HbA1c, and the risk factors on CAVI, the overall adjusted R^2^ of age, BMI, sex differences, and HR was 0.460, whereas FPG and HbAlc contributed to the addition of the overall adjusted R^2^ by 0.037 and 0.03, respectively. In the examination of *β* values of the independent variables, changes of one standard deviation of age resulted in changes of 0.63 standard deviations of the CAVI, while one standard deviation of FPG and HbA1c resulted in changes of 0.214 and 0.200 standard deviations of the CAVI. The effect of BMI on CAVI was greater than that of FPG and HbA1c on CAVI. Considering the addition of the predictive power of FPG and HbA1c to the overall adjusted R^2^ and their *β* values, it appears that despite the independent effect of glucose level indicators on arterial stiffness, risk factors, including aging and BMI, should be cautiously monitored, and managed along with FPG and HbA1c for the progression of arterial stiffness.

With age, the artery enlarges with wall thickening and elastic properties at the level of the large arteries are reduced (25). It has been reported that sex hormones, especially estrogen, induce differences in arterial stiffness between the sexes. During reproductive age, arterial stiffness is lower in women than in men; however, during menopause, arterial stiffness rises rapidly in women, which may be mediated by estrogen (26). However, the effect of sex differences on arterial stiffness is not consistent with previous studies in the Korean population. Shin et al. reported that sex differences were not predictive of an increase in ba-PWV (8), whereas Kim et al. reported a significant increase in cf-PWV in men than in women (27). This study supports Kim’s study results, and the effect of sex hormones on the increase in arterial stiffness may not have been activated, so CAVI was lower in women than in men (27). Although a significant positive correlation was observed between the degree of obesity and PWV (19), several studies on the relationship between BMI and arterial stiffness reported negative correlations between them (11, 28, 29). This study also found that a decrease in BMI was predictive of an increase in CAVI. Concerning the “obesity paradox,” Tang et al. speculated that differences in sample size, interaction with BMI and other risk factors such as BP, differences in metabolic effects between obese and non-obese groups, and differences in waist/hip ratio and BMI as an obesity indicator, may have resulted in negative correlations between obesity indicators and arterial stiffness (29). In this study, the mean BMI value of men was 24.97 kg/m^2^ and that of women was 23.28 kg/m^2^. Therefore, one possibility is that the relationship between BMI and CAVI in the non-obese group may have been different from that in the overweight or obese group. Another possibility is that the interaction between age and BMI may have contributed to the “obesity paradox”. Hence, BMI may have decreased with age among the participants of this study. However, the examination of the interactions between risk factors and glucose level indicators was not conducted in this study, and it is challenging to examine the “obesity paradox,” considering the differences between obese and non-obese groups and interactions between BMI and other risk factors, in further studies.

Since a strong age effect was found through regression analysis, partial correlations between CAVI and the SDPTG indices with age as a covariate were conducted and compared using Spearman’s correlations. Spearman’s correlations showed moderate levels (r; 0.319 -0.410) and they were higher than the correlations between cf-PWV and SDPTG indices in Hashimoto’s study (r; 0.164-0.205) (30). However, partial correlations were much lower than Spearman’s correlations, and the relationship between the b/a index and CAVI became non-significant. Considering hierarchical regression analysis and lowered correlations after correcting for age, SDPTG indices may not possibly be involved in the mechanism by which blood glucose levels affect arterial stiffness, although CAVI and SDPTG may share the mechanism by which risk factors such as age, sex, and BMI affect arterial stiffness. Zheng et al. speculated that increased blood glucose levels may affect arterial stiffness through damage to endothelial and capillary diastolic functions (31). Arterial stiffness may again decrease capillary perfusion so that insulin resistance in hepatic tissue or skeletal muscle and blood glucose levels will be increased. Therefore, one possibility is that CAVI may be more sensitive to damage to the endothelial wall and decreased capillary perfusion due to increased glucose levels than SDPTG. Interestingly, one study reported that the b/a index was higher in the diabetes group with over 8.0% of HbA1c than in the diabetes group under 8.0% of HbA1c (14). Considering that b/a reflects large arterial stiffness while d/a reflects peripheral artery stiffness (12), and that most subjects (259/276 by FPG, 256/276 by HbA1c) were included in the normal and prediabetes groups, it is still possible that b/a may reflect large arterial stiffness due to increased glucose levels in the advanced stage of diabetes.

In the examination of differences in CAVI among the four groups by HbA1c, CAVI was lower in the low-normal, high-normal, and prediabetes groups than in the diabetes group. This indicated that despite the linear relationship between blood glucose levels and arterial stiffness, there was a possibility of rapid aggravation of arterial stiffness at the beginning of diabetes. CAVI was higher in men than in women in all four groups. However, differences in CAVI among the four groups by FPG were rearranged into three groups: CAVI in the high-normal and prediabetes groups was higher than that in the low-normal group, and CAVI in the diabetes group was higher than that in the high-normal and prediabetes groups. It is not clear why this discrepancy occurred between the FPG and HbA1c levels. One possibility is that FPG values were not normally distributed; therefore, the assignment of sample numbers to each group by FBS was not as homogeneous as that by HbA1c. Therefore, further studies including more samples from patients with diabetes are needed to clarify the changes in CAVI.

Kruskal−Wallis rank and Mann−Whitney U tests showed that d/a in the high-normal and prediabetes groups was lower than d/a in the low-normal group; thereafter, d/a increased in the diabetes group, equivalent to that in the low-normal group. This indicated that peripheral artery stiffness estimated by d/a increased according to increased glucose levels in the high-normal and prediabetes stages, and again decreased in the diabetes stage. However, this result should be interpreted cautiously because hierarchical regression models did not find a significant relationship between FPG and d/a. Therefore, it does not appear that increased glucose levels directly result in changes in d/a. One possibility is that the compensation mechanism may have been activated during the pre-diabetes stage. Increased glucose levels induce endothelial vasodilator dysfunction (29). Insulin itself vasodilates skeletal muscle so that insulin-induced microvascular flow increases its tissue delivery, as well as the delivery of nutrient substrates in the prediabetes stage (32). After entering the diabetes stage, endothelial damage due to increased insulin resistance may have a more dominant influence on arterial stiffness than the compensation mechanism of peripheral vasodilation.

This study has some limitations. The sample size of the diabetes group was smaller than those of the other three groups. Data on medications for hypertension or hyperlipidemia, regular exercise, sedentary lifestyle, body composition, and smoking were not considered. Interactions among FPG, HbA1c, and other risk factors were not considered. The results of this study are limited to the Korean population only. Further studies are required to validate the results of this study.

## 5 Conclusions

This study found that none of the SDPTG indices were predicted by FPG or HbA1c levels, although other risk factors such as age, sex, and BMI were significant predictors of the SDPTG indices. Hence, CAVI may not be substituted for SDPTG indices to estimate arterial stiffness with increased glucose levels possibly because SDPTG indices may not reflect the mechanism by which blood glucose affects arterial stiffness. Moreover, the progression rate of arterial stiffness may differ between the diabetic and nondiabetic stages.

## Data availability statement

The original contributions presented in the study are included in the article/**Supplementary Material**. Further inquiries can be directed to the corresponding author.

## Ethics statement

The studies involving humans were approved by the Institutional Review Board of the Kyung Hee University Korean Medical Hospital at Gangdong. The studies were conducted in accordance with the local legislation and institutional requirements. The ethics committee/institutional review board waived the requirement of written informed consent for participation from the participants or the participants’ legal guardians/next of kin for this retrospective study under the premise that all personal information was concealed.

## Author contributions

Study concept and design, acquisition of data, analysis and interpretation of data, drafting of the manuscript, statistical analysis: Y-JP.
